# Evaluation of the FMDV 2.1 dry RT-rtPCR prototype assay for detection of foot-and-mouth disease virus in lymph nodes

**DOI:** 10.1177/10406387261451770

**Published:** 2026-06-01

**Authors:** Khomotso C. Moabelo, Rolf Rauh, Angelika Loots, Sven D. Parsons, Melvyn Quan

**Affiliations:** Departments of Veterinary Tropical Diseases, Faculty of Veterinary Science, University of Pretoria, Onderstepoort, South Africa; Tetracore, Rockville, MD, USA; Departments of Veterinary Tropical Diseases, Faculty of Veterinary Science, University of Pretoria, Onderstepoort, South Africa; Anatomy and Physiology, Faculty of Veterinary Science, University of Pretoria, Onderstepoort, South Africa; (Parsons); Deltamune, Centurion, South Africa; Departments of Veterinary Tropical Diseases, Faculty of Veterinary Science, University of Pretoria, Onderstepoort, South Africa

**Keywords:** *Aphthovirus vesiculae*, cattle, homogenization, RT-rtPCR, pen-side, point-of-care assay

## Abstract

We evaluated the performance of the FMDV 2.1 dry RT-rtPCR prototype assay (Tetracore) to detect foot-and-mouth disease virus (FMDV) in lymph nodes. Various masses (20–100 mg) of FMDV-negative lymph node tissue were homogenized, diluted, and spiked with FMDV RNA to determine the optimal amount for detection. Optimal amplification was obtained using 20 mg of tissue. Fifteen lymph nodes collected from animals challenged with FMDV that had clinical signs were subjected to manual and automated homogenization and tested using the FMDV 2.1 dry RT-rtPCR prototype assay on a T-COR 8 thermocycler (Tetracore). Additionally, automated homogenates were tested using the World Organisation for Animal Health (WOAH)-recommended RT-rtPCR assay on a CFX real-time thermocycler (Bio-Rad). Strong Pearson correlation was observed between homogenization methods using the FMDV 2.1 dry RT-rtPCR assay (*r* = 0.74, 95% CI [0.49, 1.00]; *n* = 15, *p* < 0.001). The Pearson correlation between the 2 RT-rtPCR assays was weak (*r* = 0.25, 95% CI [−0.78, 1.00]; *n* = 15, *p* = 0.076). When manual and automated homogenization methods were compared using the FMDV 2.1 dry RT-rtPCR prototype assay, 6 of the 15 samples tested positive, 7 tested negative, and 2 had a discrepant result. When the samples homogenized using the automated method were subsequently tested with both the FMDV 2.1 dry RT-rtPCR prototype assay and the WOAH-recommended RT-rtPCR assay, 5 of the 15 samples were positive, 5 were negative, and 5 were discrepant. The Cohen kappa coefficient indicated substantial agreement between homogenization methods (0.74) and fair agreement between assays (0.36). Our findings provide preliminary support for the FMDV 2.1 dry RT-rtPCR prototype assay, but the assay requires further optimization.

Foot-and-mouth disease virus (FMDV; family *Picornaviridae*, taxon species *Aphthovirus vesiculae*) is the cause of the highly infectious foot-and-mouth disease (FMD). The infection affects cloven-hoofed animals, and outbreaks can result in significant global economic consequences.^1^ Rapid and accurate virus identification is crucial for disease control.^9,15^ The traditional diagnosis of FMD by clinical assessment supported by serologic assays may not be sufficiently sensitive in the initial stages of infection.^13^

Surveillance for FMDV faces significant challenges given the complex pathogenesis and tissue distribution patterns of the virus. In infected cattle, FMDV rapidly concentrates in lymph nodes draining the pharyngeal region, particularly the medial and lateral retropharyngeal nodes, where it can persist for extended periods.^11^ Remnants of viral genome and proteins can remain detectable in these nodes for >1 mo post-infection; most non-pharyngeal tissues typically clear the virus within 4–14 d, making lymphoid tissues a sensitive and practical indicator of recent or ongoing FMDV infection. Moreover, intact viral particles can be retained in lymph node germinal centers by follicular dendritic cells for up to 38 d, even when undetectable in surrounding tissues.^12,18^ Although advanced molecular platforms, such as the T-COR 8 real-time PCR thermocycler (Tetracore), offer rapid detection capabilities, they are not yet validated for use with lymph node tissue.

The rapid turnover time, high sensitivity, and specificity of quantitative real-time PCR (qPCR) assays have revolutionized the detection of animal viruses.^3^ However, in the field or in resource-limited environments in which timely diagnosis is critical for controlling an outbreak, qPCR assays may be less useful. These tests typically rely on laboratory facilities and skilled personnel, which may not be close to where the samples are collected. Delays also can occur when shipping samples to the laboratory. Furthermore, the high cost and infrastructure requirements restrict the use of qPCR to well-equipped laboratories, rather than in the field.^7^

Several portable detection assays—such as the FMDV 2.0 dry reverse-transcription quantitative real-time PCR (RT-rtPCR) assay (Tetracore), which is run on the T-COR 8 thermocycler—have emerged as promising solutions to mitigate the drawbacks of traditional laboratory systems. The rapid and accurate diagnosis of FMD in remote regions is made possible using portable instruments. Designed for rapid, field-based testing, the T-COR 8 system can detect FMDV in ~90 min, which makes decisions more timely during outbreaks in resource-limited environments.^2,10^ On-site testing can significantly improve response times and disease control measures.^9,16^

We evaluated the potential inhibitory effects of lymph node tissue matrices on the performance of the FMDV 2.1 dry RT-rtPCR prototype assay using the T-COR 8 thermocycler, determined the optimal tissue mass, and compared manual and automated homogenization methods. We then compared the FMDV 2.1 dry RT-rtPCR prototype assay with a World Organisation for Animal Health (WOAH)-recommended laboratory-based RT-rtPCR assay for detecting FMDV^5^ (**Fig. 1**).

**Figure 1.**
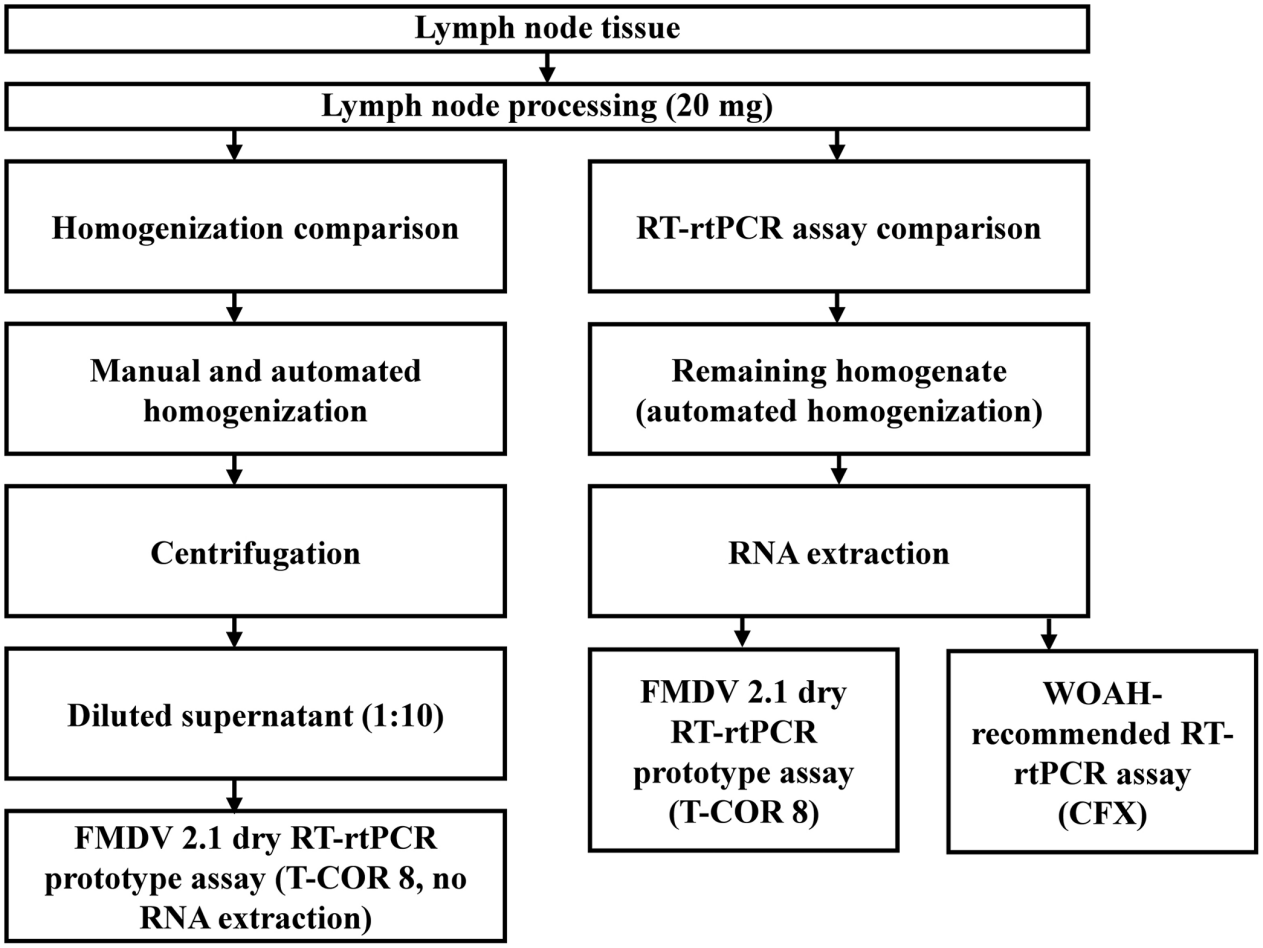
Experimental design. Lymph node tissue (20 mg) underwent both manual and automated homogenization. For the homogenization comparison, diluted supernatants were tested directly using the foot-and-mouth disease virus (FMDV) 2.1 dry RT-rtPCR prototype assay on a T-COR 8 thermocycler. The remaining homogenate (from automated homogenization only) was subjected to RNA extraction, and the extracted RNA was tested using both the FMDV 2.1 dry RT-rtPCR prototype and the World Organisation for Animal Health (WOAH)-recommended RT-rtPCR assays. The manual homogenate was not subjected to RNA extraction.

Our study was conducted at the Agricultural Research Council’s Onderstepoort Veterinary Research Transboundary Animal Diseases facility (ARC OVR TAD; Pretoria, South Africa) and approved by the Research Ethics Committee of the Faculty of Veterinary Science, University of Pretoria (REC028-19, REC069-22). The Department of Agriculture, Land Reform and Rural Development approved the study to be performed under Section 20 of the Animal Diseases Act No. 35, 1984.

To determine the optimal tissue mass for amplification using the FMDV 2.1 dry RT-rtPCR prototype assay, FMDV-negative lymph node tissue obtained from a cow originating from the FMD-free zone of South Africa was tested in duplicate in various masses (20, 40, 60, 80, and 100 mg). Tissue samples were homogenized manually in PCRD buffer (Svanodip FMDV-Ag extraction; Svanova), diluted 1:10, spiked with FMDV-positive control (FMDV 2.1 dry RT-rtPCR prototype assay), and analyzed on the T-COR 8 thermocycler according to the manufacturer’s instructions. Efficient amplification of the FMDV-positive control spiked tissue samples determined the optimal tissue amount. Positive reactions were defined as those yielding a detectable Ct value.

Twenty mg of lymph node tissue yielded the most efficient RNA amplification during the detection of FMDV using the FMDV 2.1 dry RT-rtPCR prototype assay, with the lowest and consistent Ct value (30.2). Increasing tissue masses above 20 mg resulted in progressively higher Ct values (32.8 at 40 mg, 34.8 at 60 mg, and 36.6 at 80 mg), indicative of delayed PCR inhibition, with no amplification observed at 100 mg, indicating complete inhibition at higher tissue masses.

Lymph node samples were obtained from a vaccine trial conducted at ARC OVR TAD. In the trial, groups A and B were vaccinated with different vaccine formulations, and control group C was unvaccinated. Animals were challenged 56 d after vaccination with 0.2 mL of 10^4^ 50% tissue culture infectious dose (TCID_50_) of FMDV, strain KNP/1/10, injected intradermolingually. Animals were culled 15 d after challenge, and the right submandibular, prescapular, and popliteal lymph nodes (*n* = 15) were collected from 6 animals across the 3 experimental groups (**Table 1**). All lymph node samples were collected from cattle that had clinical signs consistent with FMD, including vesicular lesions affecting the tongue and all 4 feet.

**Table 1.** Animal groups, challenge virus (KNP/1/10 (0.2 mL of foot-and-mouth disease virus [FMDV] at TCID_50_ of 10^4^/mL intradermolingually), clinical signs, and sample types used in our evaluation of the FMDV 2.1 dry RT-rtPCR assay for the detection of foot-and-mouth disease virus in lymph nodes.

Group	Animal	Clinical signs		Lymph node
		Feet	Tongue	
A	1	+*	+	Right popliteal, submandibular, prescapular
	2			Right popliteal, submandibular, prescapular
B	3	+	+	Right popliteal, submandibular, prescapular
C (control)	4	+	+	Right submandibular, prescapular
	5			Right submandibular, prescapular
	6			Right submandibular, prescapular

* Indicates clinical signs observed in the specified area.

Homogenization methods were compared using 20 mg of tissue processed manually and through automated bead-based homogenization using zirconium beads (Biotechnology Hub Africa). Manual homogenization was as described above, but using 20 mg of lymph node tissue and not spiking the sample with positive control. The diluted homogenates were analyzed in duplicate on the T-COR 8 thermocycler, according to the manufacturer’s instructions. Positive reactions were defined as those yielding a detectable Ct value. For automated homogenization, 20 mg of lymph node tissue was homogenized for 90 s at 1,500 rpm (1600 MiniG tissue homogenizer; Spex SamplePrep [currently Cole-Parmer SamplePrep]) in PCRD buffer (Svanodip FMDV-Ag extraction). The homogenate was centrifuged (5415D; Eppendorf) at 14,600 ×* g* for 20 s, after which 10 μL of the supernatant was diluted with 90 μL of nuclease-free water and tested in duplicate using the FMDV 2.1 dry RT-rtPCR prototype assay on a T-COR 8 thermocycler without prior RNA extraction. Positive reactions were defined as those that gave a detectable Ct value, consistent with the criteria used for manual homogenization. The remaining homogenate was stored at −80°C for subsequent RNA extraction (QIAamp viral RNA mini kit; Qiagen). RNA extraction was performed on 140 μL of the sample, with elution yielding a final volume of 60 μL. Extracted RNA was then tested using both the FMDV 2.1 dry RT-rtPCR prototype assay and the WOAH-recommended RT-rtPCR assay. Amplification of the target RNA was performed (CFX-96 real-time system; Bio-Rad), using primers (final concentration: 500 nM) and probes (final concentration: 200 nM) as described by others,^5^ with validated reagents and parameters,^6^ and in accordance with the manufacturer’s instructions for TaqMan fast virus 1-step master mix (ThermoFisher). Each sample for the WOAH-recommended assay was tested in duplicate, and reactions were classified as positive if the Ct value was <36. Automated homogenization of tissue samples and analysis by the FMDV 2.1 dry RT-rtPCR prototype assay was compared with automated homogenization of tissue samples and analysis by the WOAH-recommended RT-rtPCR assay.

Results were recorded in Excel (v.2603; Microsoft) and analyzed with SPSS Statistics (v.30; IBM). Normality was evaluated with the Shapiro–Wilk test of normality. The level of agreement between the FMDV 2.1 dry RT-rtPCR prototype and the WOAH-recommended RT-rtPCR assays and the homogenization methods was compared with the Cohen kappa (κ). The Pearson correlation measured the strength and direction of the linear relationship between the results obtained from the 2 RT-rtPCR assays and the 2 homogenization methods.

The consistency of results between duplicate analyses was high for both homogenization methods, with duplicate runs producing concordant qualitative results (positive or negative) for most samples. A Pearson correlation test was a strong positive between the manual and automated homogenization results using the FMDV 2.1 dry RT-rtPCR prototype assay (*r* = 0.74, 95% CI [0.49, 1.00]; *n* = 15, *p* < 0.001).^14^ Hence, the manual method achieved homogenization as well as the automated method. The overall concordance between the 2 homogenization methods was 13 of 15, with a proportion of positive agreement of 0.857 and negative agreement of 0.875. The fact that only 2 of 15 test results disagreed between the homogenization methods underlines the reliability of the process as a preliminary step.

The Cohen κ coefficient between the 2 methods of 0.74 (z = 2.958; *p* < 0.003) was substantial, with a 95% CI of [0.41, 1.00]. Results were concordant between the homogenization methods, except that results were discrepant for 2 of 15 samples for automated homogenization (**Table 2**). Discrepancies may have arisen from tissue‑derived inhibitors as well as differences in homogenization intensity and settings, which could influence RT-rtPCR Ct values. Manual homogenization methods, although less prone to heat generation, may also be affected by operator–to–operator variability.

**Table 2.** Results obtained from testing 15 lymph node samples processed using manual and automated homogenization methods and tested with the foot-and-mouth disease virus (FMDV) 2.1 dry RT-rtPCR prototype assay.

Manual homogenization	Automated homogenization		Total
	Positive	Negative	
Positive	**6**	2	8
Negative	0	**7**	7
Total	6	9	15

Boldface = concordant results between homogenization methods.

For the 2 RT-rtPCR assays, the Pearson correlation had a weak positive relationship (*r* = 0.25, 95% CI [−0.78, 1.00]; *n* = 15, *p* = 0.076). The overall concordance between the FMDV 2.1 dry RT-rtPCR prototype and the WOAH-recommended RT-rtPCR assays was 10 of 15, with a proportion of positive agreement of 0.706 and a negative agreement of 0.615, suggesting variability in assay performance. The Cohen κ coefficient between the 2 assays of 0.36 (z = 1.506, 95% CI [−0.07, 0.77]; *p* = 0.132) indicated fair agreement.^17^ Both RT-rtPCR assays had discrepant results; 5 of 15 samples yielded discrepant results; 4 of 15 tested positive on the WOAH-recommended assay, whereas 1 of 15 tested positive on the FMDV 2.1 dry RT-rtPCR prototype assay (**Table 3**). Only 1 of 5 discrepant samples was the result of differences in interpretation rules, with a Ct value of 36.8 in the reference assay, which was considered negative according to its positivity threshold but positive on the FMDV 2.1 dry RT-rtPCR prototype assay. The remaining 4 discrepant samples (CFX-96, Ct ≤ 36) were true differences in assay detection and were not attributable to interpretation criteria.

**Table 3.** Results obtained from testing 15 lymph node samples using foot-and-mouth disease virus (FMDV) 2.1 dry RT-rtPCR prototype and World Organisation for Animal Health (WOAH)-recommended RT-rtPCR assays.

FMDV 2.1 dry RT-rtPCR prototype assay	WOAH-recommended RT-rtPCR assay		Total
	Positive	Negative	
Positive	**5**	1	6
Negative	4	**5**	9
Total	9	6	15

Boldface = concordant results between RT-rtPCR assays.

Most discrepant results occurred in samples with Ct values near or above the reference assay positivity threshold (Ct ≤ 36), indicating that disagreements between the 2 assays were largely confined to low-viral-load samples near the limit of detection. The observed discordance between the 2 RT-rtPCR assays (5 of 15 samples) reflects a combination of technical and biological factors. Variability in test outcomes may be influenced by differences in assay chemistry, the quality of the sample, and the efficiency of nucleic acid extraction,^4^ particularly when testing complex tissue matrices such as lymph nodes. Our inconsistent RT-rtPCR results are consistent with previous research, which has shown that the design of the primer and probe, the chemical composition of the amplification, and the target abundance all contribute to variability.^8^

Our study was limited by the small number of samples and animals evaluated, all of which were derived from clinically affected animals. Further studies incorporating larger numbers of samples, including more positive and negative samples, are required to fully validate the use of lymph nodes and the T-COR 8 platform for FMDV detection.
